# Psychological Distress and Use of Psychotropic Drugs Among University Students—the SHoT Study, Norway

**DOI:** 10.3389/fpsyt.2021.717955

**Published:** 2021-09-20

**Authors:** Ivana Bojanić, Erik R. Sund, Ottar Bjerkeset, Børge Sivertsen, Hege Sletvold

**Affiliations:** ^1^Faculty of Nursing and Health Sciences, Nord University, Levanger Municipality, Norway; ^2^Department of Public Health and Nursing, Faculty of Medicine and Health Science, HUNT Research Centre, Norwegian University of Science and Technology, Levanger Municipality, Norway; ^3^Levanger Hospital, Nord-Trøndelag Hospital Trust, Levanger Municipality, Norway; ^4^Department of Mental Health, Faculty of Medicine and Health Sciences, Norwegian University of Science and Technology, Trondheim, Norway; ^5^Department of Health Promotion, Norwegian Institute of Public Health, Bergen, Norway; ^6^Department of Research & Innovation, Helse Fonna HF, Haugesund, Norway

**Keywords:** students, mental health, psychological distress, psychotropics, antidepressants, hypnotics, anxiolytics, gender differences

## Abstract

**Background:** Students pursuing higher education are struggling with psychological distress, which in turn may negatively affect their academic self-efficacy and study progress. Although psychotropic drug use is widespread and increasing, patterns of psychotropic drug use among students are not well-known.

**Aim:** To describe prevalence and gender differences in psychotropic drug use among Norwegian students in higher education, and to examine associations with level of psychological distress.

**Methods:** The study is based on data from the Norwegian Student's Health and Well-being Study (SHoT), 2018, a national survey including all fulltime students aged 18–35 years in higher education. Our sample included 49,836 students, 69% females. Use of psychotropic drugs and psychological distress (The Hopkins Symptoms Checklist [HSCL-25]) were self-reported. Generalised linear models were used to assess associations between psychological distress and psychotropic drug use.

**Results:** Psychotropic drug use was more frequent among female than male students: 4 vs. 2% daily antidepressants usage; 5 vs. 3% last month use of anxiolytics/tranquillisers; and 8 vs. 5% last month use of hypnotics. In contrast, male students reported use of performance enhancing drugs more often than females (7 vs. 5%). Adjusted associations between high level of psychological distress (HSCL-25 ≥ 2.0) and use of psychotropics, showed an about 2-fold increased relative risk, largely consequent across drug classes and genders.

**Conclusion:** Prevalence and gender patterns of intake of the most common psychotropic drug classes among Norwegian students are comparable to previous studies. Unexpectedly, among students with moderate to severe psychological distress, the patterns of psychotropic drug use were more or less identical between genders.

## Introduction

Psychological distress is highly prevalent among university and college students worldwide (1) and often has negative effects on several domains of students' life (2, 3). Psychological distress is defined as emotional suffering characterised by symptoms like depression (e.g., loss of interest, sadness, hopelessness), anxiety (e.g., restlessness, feeling tense) and functional or somatic symptoms (e.g., headache, insomnia, poor appetite, sexual dysfunction, and lack of energy) (4). A recent literature review found that psychological distress was more prevalent and severe among university students compared to their non-studying age-matched peers in the general population (5). Furthermore, psychological distress among students is strongly associated with lower academic self-efficacy and reduced study progress (6), and increased risk of suicidal thoughts and behaviours (7). Thus, preventive interventions, early detection, and optimal treatment of mental illness among students, as well as general improvement of students' mental health, have become urgent matters.

Both non-pharmacological interventions and psychotropic drugs are cornerstones in the treatment of mental illnesses, and women are more frequent users of both mental healthcare services and these drugs, compared to men (8, 9). A psychotropic drug can be defined as any drug affecting the mind, emotions and behaviour, e.g., stimulants, antidepressants, antipsychotics, anxiolytics, and hypnotics. Parallel to the striking increase in psychological distress in adolescents and young adults (10, 11), the use of psychotropic drugs among children and adolescents has increased in many countries in the last decade (12–16). The same trends have been observed in young adults. According to the Norwegian Prescription Database (NorPD), the 1-year prevalence of psychotropic drug use has increased by about 29% in the last decade among 20–24 year-olds, to 14% in females and 9% in males, in 2019 (17). A case study from Australia confirmed an increasing trend in psychotropic drug prescribing to patients aged 16–24 year with mental disorders from 2009 to 2014, with higher prescription levels in women compared to men (18). Similarly, mental distress among Norwegian female medical students increased from 1993 to 2015, paralleled with a significant increase in mental health help-seeking (19).

Studies investigating psychotropic drug use among students in relation to psychological distress are limited. A study of 21,771 students at a US university, reported increased prevalence of past-year medical use of stimulants (from 1.9 to 4.7%), sedatives/anxiolytics (from 2.9 to 4.3%), and hypnotics (from 2.9 to 3.2%), in the 10-year period from 2003 to 2013 (20). Further, a cross-sectional national study among nearly 11,000 French medical students reported that 12% of students received psychiatric or psychological treatment, while 5.7% used anxiolytics regularly, 2.8% used antidepressants, and 2.1% were hypnotic medication consumers (21). Despite an increasing trend in help-seeking and use of psychotropics among young adults, studies have reported undertreatment of mental health disorders among students (3, 22).

Non-medical use, defined as use without a prescription or use outside the indication of the medication (23), and misuse or illicit use of psychotropic drugs among students is well-documented (24–28). A meta-analysis reported an overall rate of stimulant medication misuse among college students of 17%, more frequent among men compared to women (24). Motives for prescription drug misuse among students include personal performance enhancement in academic or sport outcomes, mental health reasons including ability to sleep or to reduce anxiety, or manage pre-existing illnesses (29). Additionally, the use of stimulants increases with stress (30) and difficulties in academic achievement (31). Moreover, the trends of increased use of prescribed psychotropic drugs among students parallel the increased non-medical use of the same psychotropics (20), highlighting the importance of monitoring prevalence of psychotropic drug use among students. The correct use of psychotropics to enhance mental health of students is of great importance, in addition to ensuring patient safety in this population.

Usually, young adults become fully responsible for their medication management for the first time in life as they become college or university students. Although the study years represent a free and liberating period in life for many young adults, a large portion of students experience student life as mentally stressful and demanding and report high levels of psychological distress (5, 11). Previous research demonstrate that psychological distress affects students worldwide (1), recognising this as an important public health issue. However, studies based on large, nationwide samples including students across all faculties and universities that allow investigation of psychological distress in relation to the use of psychotropic drugs are still scarce. The SHoT study is a national health and well-being survey among Norwegian university students, and was conducted in 2010, 2014, and 2018 (32).

The aim of this study was to study prevalence and gender differences of psychotropic drug use among Norwegian university students based on SHoT 2018 data, and to examine associations with psychological distress symptom load.

## Materials and Methods

### Study Sample and Setting

The SHoT study is a national student health survey for higher education conducted every 4 years since 2010. The size and scope of the SHoT studies has expanded over time, and this study is based on SHoT 2018, which was a collaboration between the Norwegian Institute of Public Health (NIPH) and the three largest student welfare organisations in Norway. All Norwegian fulltime students aged 18–35 years taking higher education (both in Norway and abroad) were invited for participation (*N* = 162,512). Totally, 50,054 students (31% response) completed the electronic questionnaire. SHoT 2018 includes self-reported information on mental and physical health, quality of life, health related behaviours, medication use, study related information, demographics, and background factors (33). The sample of this study includes responses from 34,437 female and 15,399 male students, excluding 218 students reporting transgender sex, giving a final sample of 49,836 higher education students. The response rate among females and males was 36.9 and 22.8%, respectively.

### Ethics

The SHoT 2018 study was approved by the Regional Committee for Medical and Health Research Ethics in Western Norway (reference number 2017/1176). Participation was voluntary, and electronic informed consent was obtained from all participants (32).

### Measurements

#### Psychological Distress

Psychological distress symptoms were assessed using The Hopkins Symptoms Checklist (HSCL-25). HSCL-25 is a widely used, validated screening tool used to assess symptoms of anxiety and depression. The scale consists of 10 items for anxiety and 15 items for depression, each scored on a Likert scale from 1 (not at all) to 4 (extremely) (34). The symptoms of anxiety and depressions were assessed for the period of the past 2 weeks. HSCL-25 has psychometric properties suitable for detecting symptoms of anxiety and/or depression in different population settings such as the general population (35), primary care outpatients (36), and refugee patients with different cultural backgrounds (37). To indicate clinically relevant psychological distress symptom load, a cut-off at 1.75 was proposed in the original version (34) and used in several studies (11, 36). Additionally, a more conservative cut-off of ≥2.0 has been used to indicate moderate to severe psychological distress symptoms (6, 32). This study uses ≥2.0 cut-offs to describe the study population and in the logistic regression analyses.

#### Psychotropic Drug Use

The use of drugs was assessed by asking if the participants had used either of the following medicines at least once a week during the last 4 weeks: antidepressants, anxiolytics/tranquillisers, or hypnotics. The use of prescription analgesics, non-prescription analgesics, anti-allergic agents, antibacterial agents, anti-asthmatic agents, and any other medicine was also self-reported in the SHoT 2018 questionnaire and was combined and assessed as “any medicine” in our analysis. Response options were: *Daily*; *every week, but not daily*; *less than every week*; *not used last 4 weeks*. For antidepressants and “any medicine,” the responses were analysed as: Daily use; Used last 4 weeks, but less than daily (combining *every week, but not daily* and *less than every week*); and *Not used last 4 weeks*. For anxiolytics/tranquillisers, and hypnotics, the response were analysed as: *Used last 4 weeks*; *Not used last 4 weeks*. Regarding performance-enhancing drugs, the participants were asked: *During your study period, have you ever used drugs that increase your ability to concentrate, your alertness, increase your energy, or to calm down before exams/presentations?* The possible responses were *yes* or *no*. If answering *yes*, the following question was: *Which drug(s) have you used to increase your ability to concentrate, your alertness, increase your energy, or to calm down before exams/presentations?* The questionnaire included a list of possible drug names, for which participants could tick one or more options: *amphetamine; betablockers; donepezil; energy drinks; caffeine tablets; methylphenidate; modafinil; paracet (acetaminophen)/ibuprofen etc; other*. The number of study participants ticking the stimulants amphetamine, methylphenidate, and modafinil, were summarised.

#### Demographic and Health Characteristics

Included variables on demographic characteristics were: sex, age (continuous variable), relationship status (coded as *single*; partner, lives alone; *married, cohabitant*), live alone (coded as *yes* or *no*), foreign-born (coded as *yes* or *no*). Financial distress was assessed by the question: “*For most of the last 12 months, was your household's finances so that you would manage an unforeseen bill of NOK 5,000?”* (coded as *yes* or *no*). For details regarding the financial distress variable, see Bøe et al. (38). Loneliness was assessed by the question *How often do you feel isolated from others*?, where the students were asked to indicate how often they felt that way during the last year, on a 5-point Likert scale; *never, seldom, sometimes, often, very often*. For details, see Hysing et al. (39). Self-rated global health was assessed by asking the students to rate their own health with the possible answers *poor, fair, good, excellent*.

### Statistical Analysis

Difference in self-reported psychological distress was examined by comparing total HSCL-25 score [mean (95% confidence interval (CI)] as well as proportions (95% CI) scoring above the cut-offs between the groups in relation to health-related factors, medication use, lifestyle, background information, sociodemographic characteristics, and clinical measurements. Generalised linear models with a log link function and identity link function were used to calculate rate ratio (RR) and rate difference (RD) between psychotropic drug use and self-reported psychological distress, respectively. The models were adjusted for age, age squared, financial distress, living alone, self-rated health, loneliness, marital status, and foreign-born variables. We obtained robust standard errors for the parameter estimates. RR and RD are reported with 95 per cent confidence intervals (95% CI). Stata v.16 was used for all data management and statistical analysis (40).

## Results

Table 1 shows the demographic and health characteristics of the university student population, including psychological distress symptom load, gender stratified. Two thirds of students were female, and the mean age was 23.1 and 23.5 years among women and men, respectively. There were 7,661 foreign-born study participants, 14.9% of the females and 16.5% of males. Half of the study participants were single, and 19.3% lived alone. Nearly 17% of the females and 15% of the males felt lonely often or very often. Furthermore, 58.9% of the women, and 67.7% of the men reported financial distress. Further, 23.1% of the females and 14.9% of the males reported “poor” or “not so good” global health. The prevalence of moderate to severe symptom load of anxiety and depression (HSCL-25 ≥ 2.00) was 33.6% in female and 16.8% and male students.

**Table 1:** Demographic and health characteristics of the student population, gender stratified.

		**Females** ***n*** **= 34,437**		**Males** ***n*** **= 15,399**	
		***n***	**%**	***n***	**%**
**Marital status (** ***n =*** **49,764)**					
	Single	16,238	47.2	8,617	56.0
	Partner, lives alone	8,258	24.0	3,582	23.3
	Married, cohabitant	9,896	28.7	3,173	20.6
**Living alone (** ***n =*** **49,836)**					
	No	27,992	81.3	12,210	79.3
	Yes	6,445	18.7	3,189	20.7
**Foreign-born/Born outside Norway (** ***n =*** **49633)**					
	No	29,175	84.7	12,797	83.1
	Yes	5,128	14.9	2,533	16.5
**Financial distress (** ***n =*** **49,603)**					
	No	13,960	40.5	4,922	32.0
	Yes	20,291	58.9	10,430	67.7
**Loneliness (** ***n =*** **49,198)**					
	Never	9,775	28.4	5,271	34.2
	Seldom	10,619	30.8	4,714	30.6
	Sometimes	7,866	22.8	2,954	19.2
	Often	3,837	11.1	1,459	9.5
	Very often	1,997	5.8	797	5.2
**Self-rated global health (** ***n =*** **49,751)**					
	Poor	891	2.6	264	1.7
	Fair	7,071	20.5	2,023	13.1
	Good	17,138	49.8	7,359	47.8
	Excellent	9,284	27.0	5,721	37.2
**Psychological distress (** ***n =*** **49,730)**					
HSCL-25^*^	<2.00	22,811	66.2	12,742	82.7
	≥2.00	11,585	33.6	2,592	16.8
**Age (** ***n =*** **49,157)**		Mean	SD	Mean	SD
	Continuous	23.1	3.3	23.5	3.3

* *HSCL-25, The Hopkins Symptoms Checklist*.

Table 2 describes the self-reported psychotropic drug use. Female students had a higher prevalence of daily antidepressants usage (4%) than male students (2.3%). The female students' usage of anxiolytics/tranquillisers and hypnotics the last 4 weeks (4.6 and 7.7%, respectively) were higher than among males (3.2 and 5.2%, respectively). The prevalence of performance-enhancing drug use was higher among male than female students (6.5 vs. 4.8%). Totally 988 (2%) students reported to have used amphetamine, methylphenidate, and/or modafinil during their studies. The percent of missing data on psychotropic drug use variables varied between 13.2 and 14.2.

**Table 2:** Prevalence of psychotropic drug use among the student population, gender stratified.

		**Females** ***n*** **= 34,437**		**Males** ***n*** **= 15,399**	
		***n***	**%**	***n***	**%**
**Antidepressants (** ***n =*** **43,017)**					
	Daily use	1,364	4.0	361	2.3
	Used last 4 weeks, but less than daily	186	0.5	105	0.7
	Not used last 4 weeks	28,403	81.9	12,784	83.0
**Anxiolytics/tranquillisers (** ***n =*** **42,900)**					
	Used last 4 weeks	1,590	4.6	496	3.2
	Not used last 4 weeks	28,104	81.6	12,710	82.5
**Hypnotics (** ***n =*** **43179)**				
	Used last 4 weeks	2,674	7.7	797	5.2
	Not used last 4 weeks	27,226	79.1	12,483	81.1
**Performance-enhancing drugs during studies (** ***n =*** **49,563)**					
	Yes	1,660	4.8	1,001	6.5
	No	32,626	94.7	14,276	92.7
**Any medicine (** ***n =*** **44,102)**					
	Daily use	7,759	22.5	1,161	7.5
	Used last 4 weeks, but less than daily	2,187	6.4	701	4.6
	Not used last 4 weeks	20,755	60.3	11,539	74.9

Table 3 shows the adjusted associations between psychological distress symptoms and use of psychotropics. The risk of daily use of antidepressants was more than 2-fold increased among both genders, with RR 2.36 and 2.09, and RD 4 and 3.4 percentage points (pp) for males and females, respectively. Psychological distress increased the risk of using anxiolytics/tranquillisers, with a RR of 2.8 and 2.6 in male and female students, respectively, and an absolute RD of 6.5 and 5.3 pp in males and females, respectively. Furthermore, when reporting psychological distress, the risk of daily use of hypnotics increased with approximately a 2-fold in females, and 2.2 times in males.

**Table 3:** Adjusted^a^ associations between psychological distress and daily psychotropic drug use, given by risk ratio (RR) and risk difference (RD) with 95 per cent confidence interval (95% CI).

			**Relative and absolute differences^a^**			
			**RR**	**95% CI**	**RD**	**95% CI**
**Females and Males^b^**						
	Antidepressants					
		HSCL ≥ 2.0	2.16	(1.91–2.45)	0.036	(0.030–0.042)
	Anxiolytics/tranquillisers					
		HSCL ≥ 2.0	2.61	(2.34–2.92)	0.055	(0.049–0.062)
	Hypnotics						
		HSCL ≥ 2.0	2.03	(1.87–2.21)	0.068	(0.060–0.076)
	Performance-enhancing drugs					
		HSCL ≥ 2.0	1.90	(1.72–2.09)	0.039	(0.033–0.045)
	Any medicine					
		HSCL ≥ 2.0	1.00	(0.96–1.05)	0.003	(−0.008 to 0.014)
**Females**	Antidepressants					
		HSCL ≥ 2.0	2.09	(1.82–2.40)	0.034	(0.028–0.040)
	Anxiolytics/tranquillisers					
		HSCL ≥ 2.0	2.60	(2.28–2.96)	0.053	(0.046–0.060)
	Hypnotics						
		HSCL ≥ 2.0	1.99	(1.82–2.19)	0.066	(0.057–0.075)
	Performance-enhancing drugs					
		HSCL ≥ 2.0	1.92	(1.70–2.17)	0.034	(0.028–0.041)
	Any medicine					
		HSCL ≥ 2.0	1.00	(0.95–1.05)	0.001	(−0.012 to 0.013)
**Males**	Antidepressants					
		HSCL ≥ 2.0	2.36	(1.78–3.13)	0.040	(0.028–0.053)
	Anxiolytics/tranquillisers					
		HSCL ≥ 2.0	2.81	(2.23–3.55)	0.065	(0.050–0.080)
	Hypnotics						
		HSCL ≥ 2.0	2.21	(1.84–2.66)	0.076	(0.057–0.094)
	Performance-enhancing drugs					
		HSCL ≥ 2.0	1.87	(1.58–2.21)	0.056	(0.039–0.073)
	Any medicine					
		HSCL ≥ 2.0	1.06	(0.90–1.24)	0.006	(−0.013 to 0.025)

a *HSCL <2.0 was used as reference in all analyses, with RR = 1 and RD = 0. Adjusted for age, age squared, financial distress, living alone, self-rated health, loneliness, marital status, and foreign-born*.

b *Also adjusted for sex*.

## Discussion

### Main Findings

This large, nation-wide study of more than 48,000 Norwegian students from all faculties and universities, provide information on psychotropic drug usage among students in relation to psychological distress, previously scarcely described in the literature. The prevalence of psychotropic drug use was mainly in line with results of previous studies (20, 21). Both female and male students typically used antidepressants daily, while anxiolytics/tranquillisers and hypnotics were used more sporadically, which is according to clinical guidelines (41, 42). Female students were more frequent users of antidepressants than males (daily use in 4 vs. 2%), anxiolytics/tranquillisers (5 vs. 3% last month) and hypnotics (8 vs. 5% last month), while the opposite was reported for stimulants (5 vs. 7%). Overall, these results align with previous research on gender differences in mental health and help-seeking (8, 9). In contrast, within cases (HSCL cut-off at ≥ 2.0) patterns of psychotropic drug use were very similar in males and females, and they were about twice as likely to take any of the psychotropic drug classes, compared to controls (HSCL <2.0).

### Patterns of Psychotropic Drug Use in Female and Male Students

This study describes a student population where approximately one quarter of the respondents showed a moderate to severe symptom load of anxiety and depression, and a previous study has concluded that both the level and increasing prevalence of psychological distress among Norwegian students is worrying (11). We found that the prevalence of psychotropic drug use varied between genders and classes of psychotropic medications. Similar results regarding a general increase, and gender and psychotropic drug class patterns have also been reported among children and adolescents (12–16). In particular, an increased use of hypnotics and antidepressants in adolescents has received attention (12, 13, 16). Of note, the increased hypnotics usage can largely be attributed to the increased use of melatonin (12, 13, 15), which until 2020 was a prescription drug in Norway. Previous studies have emphasised that sleep problems among students are prevalent and increasing (33), and insomnia prevalence is considerably higher in university students compared to the general population (43). Therefore, continued monitoring of hypnotics use among students is warranted, with special focus on melatonin use and effects in this population.

This study describes a prevalence of performance-enhancing drug use, including stimulants the last month, of 4.8 and 6.5% among females and male students, respectively. We cannot conclude whether these drugs were illicit or misused drugs. However, a previous study described that a substantial proportion of the Norwegian students have tried illicit drugs, and males more so than females (28). A meta-analysis described the misuse of stimulant medications among students as a prevalent and growing problem. Also, being male was associated with misuse of stimulants, and the most common source of stimulants access were fellow students with prescriptions (24). McCabe et al. found that the trend of increased use of prescribed psychotropics among students was related to the increased non-medical use of these medications (20). Altogether, these results confirm the need of continued monitoring prevalence of psychotropic drug use among students and highlight the need of safeguarding the correct use of psychotropics, in addition to ensuring patient safety strategies in this population. For example, health services with interprofessional healthcare teams should be accessible to all students, being available and competent of giving advice and counselling of proper psychotropic drug use. Educational interventions on psychotropic drug use targeted at students, student counsellors, and other relevant university staff could be useful as preventive measures.

### Psychotropic Drug Use in Male and Female Students With High (HSCL ≥ 2.0) Symptom Level

Mental health help seeking is more common in female compared to male students (6), and previous studies of young adults with mental health conditions have confirmed that females are more likely to receive treatment than males (3, 18). Brijnath et al. described an increasing trend of general practitioners' prescription of psychotropics to young people with mental health conditions from 2009 to 2014, and females had a higher incident rate for prescription than males (IRR 1.15, 95% CI 1.08–1.22) (18). In the study by Bruffaerts et al., the self-reported receipt of mental health treatment was examined among nearly 14,000 first year students from 19 colleges in eight countries world-wide. They reported on sociodemographic predictors of treatment, where being female was an independent predictor of both lifetime and 12-month treatment (3). In our study, however, the use of psychotropic drugs among female and male students with psychological distress was more or less identical across all psychotropic drug classes.

Evidently, help-seeking involves opportunities for diagnosis and treatment, and implies positive association to medication usage. However, several factors are known to affect the use of mental healthcare among young adults, e.g., type and severity of mental health problem, stigma, mental health literacy, socioeconomic position, healthcare organisation and infrastructure (8, 44), that would subsequently influence possibilities for detection of treatment needs. Furthermore, help-seeking among students is generally low, varies between university and college campuses, and across student characteristics (6, 45, 46). Accordingly, a high unmet need for treatment of mental disorders among students has been recognised (3, 22). Further research regarding psychotropic drug use among students with mental health problems is warranted, and should involve longitudinal studies, and examine gender differences of causal and treatment factors, as well as medication safety issues in this population. Furthermore, systematic strategies to increase detection of need for mental health treatment among university students is of great importance, and might need several interventions at different levels, including an interdisciplinary approach. Generally, health authorities and universities need to focus on student mental health, to reduce psychological distress among students, and encourage students to seek help when needed.

### Strengths and Limitations

The main strength of this study is the large, national sample of university students.

Limitations include the modest response rate (31%) and the potential selection bias regarding the prevalence measures and associations (32). Furthermore, self-reported anxiety and depression symptoms were assessed using the HSCL-25 with a conventional cut-off at ≥2.0. Although commonly used, and often the only feasible method in large scale studies, self-reported symptom levels do not compare directly to diagnostic categories of these conditions. Similarly, the self-reported use of medication has obvious limitations compared to register data, and the SHoT 2018 survey did not include information on whether the psychotropics were obtained through a prescription. The self-reported psychotropic drug usage was reported as daily or monthly prevalence, and are thereby not directly comparable to past-year prevalence commonly used in other pharmacoepidemiologic studies.

## Conclusion

Our study suggests similar prevalence and gender patterns of intake of the most common psychotropic drug classes among Norwegian students, compared to European and US studies (20, 21). The vast majority of antidepressant users took their drugs daily, according to the prescription guidelines (41). In contrast, anxiolytics and sedatives were taken more infrequently, which is also recommended. Although mental health help seeking is much more common in female compared to male students (6, 19), the patterns of drug use in female and male students were more or less identical across all psychotropic drug classes in our study.

## Data Availability Statement

The datasets for this article are not publicly available because of privacy regulations from the Norwegian Regional Committees for Medical and Health Research Ethics (REC). Requests to access the datasets should be directed to BS (borge.sivertsen@fhi.no). Guidelines for access to SHoT data are found at https://www.fhi.no/en/more/access-to-data. Approval from REC (https://helseforskning.etikkom.no) is a pre-requirement.

## Ethics Statement

The studies involving human participants were reviewed and approved by Regional Committee for Medical and Health Research Ethics in Western Norway (reference number 2017/1176). The patients/participants provided their written informed consent to participate in this study.

## Author Contributions

IB and ES conducted analyses. IB and HS drafted the manuscript. All authors contributed to conception of the study, interpretation of the findings, and critical revisions of the manuscript for intellectual content and have approved the manuscript for submission.

## Funding

SHoT2018 has received funding from the Norwegian Ministry of Education and Research (2017) and the Norwegian Ministry of Health and Care Services (2016).

## Conflict of Interest

The authors declare that the research was conducted in the absence of any commercial or financial relationships that could be construed as a potential conflict of interest.

## Publisher's Note

All claims expressed in this article are solely those of the authors and do not necessarily represent those of their affiliated organizations, or those of the publisher, the editors and the reviewers. Any product that may be evaluated in this article, or claim that may be made by its manufacturer, is not guaranteed or endorsed by the publisher.
